# Proton nuclear magnetic resonance and pattern recognition analysis of liver extracts from rats under different anesthetics

**DOI:** 10.1186/1471-2342-12-28

**Published:** 2012-08-16

**Authors:** Tomoyuki Tajima, Keiko Hirakawa, Hiroshi Kawaguchi, Atsuhiro Sakamoto

**Affiliations:** 1 Department of Anesthesiology, Nippon Medical School, Bunkyo-ku, Tokyo, Japan; 2 NMR Laboratory and Department of Legal Medicine, Nippon Medical School, Bunkyo-ku, Tokyo, Japan

## Abstract

**Background:**

Although general anesthesia is widely used in the surgical arena, the mechanisms by which general anesthetics act remain unclear. We previously described alterations in gene expression ratios in hepatic tissue taken from rats treated with anesthetics. Consequently, it is considered that anesthetics influence liver metabolism. Thus, the goal of this study was to use pattern recognition analysis of proton nuclear magnetic resonance spectra to visualize changes in liver metabolic phenotypes in response to widely used intravenous anesthetics (propofol and dexmedetomidine) and inhalational anesthetics (sevoflurane and isoflurane).

**Methods:**

Rats were randomized into 13 groups (n = 6 in each group), and each group received one of following agents: propofol, dexmedetomidine, sevoflurane, isoflurane, or no anesthetic (control group). The liver was directly removed from rats immediately after or 24 h or 48 h after a 6-h period of anesthesia. Hydrophilic compounds were extracted from the liver and were analyzed with proton nuclear magnetic resonance spectroscopy. All spectral data were processed and analyzed by principal component analysis for comparison of metabolite profiles.

**Results:**

Data were visualized by plotting principal component (PC) scores. In the plots, each point represents an individual sample. Each group was clustered separately on the plots, and the PC scores of the propofol group were clearly distinct from those of the control group and other anesthetic groups. The difference in PC scores was more pronounced immediately after completion of anesthesia when compared with 24 or 48 h after completion of anesthesia. Although the effect of intravenous anesthetics on the liver dissipated over time, the effect of inhalational anesthetics persisted.

**Conclusions:**

Propofol, dexmedetomidine, sevoflurane and isoflurane exert different effects on liver metabolism. In particular, liver metabolism was markedly altered after exposure to propofol. The effect of anesthesia on the liver under propofol or dexmedetomidine resolved rapidly when compared with the effect under sevoflurane or isoflurane.

## Background

General anesthesia is widely used in the surgical arena, with good efficacy and safety demonstrated for a variety of agents, including sevoflurane [1], isoflurane [2], propofol [3], and dexmedetomidine [4]. However, the mechanisms by which these agents act remain unclear. We previously reported that anesthetic agents altered the gene expression ratios in rat livers [5], which suggest that these agents can influence hepatic metabolism.

Sevoflurane and isoflurane are inhalational anesthetics. They have low solubilities in blood and tissues (resulting in rapid recovery from anesthesia), and are frequently used. Propofol is the most commonly used intravenous anesthetic and is administered as an alkylphenol formulated in a lipid emulsion [6]. Propofol provides rapid and smooth induction of anesthesia and exhibits rapid clearance from the body. Dexmedetomidine is an ideal intravenous anesthetic. It is a highly selective α_2_-adrenergic agonist, produces sedation, hypnosis, and analgesia, and has a minimal effect on respiration. Dexmedetomidine is rapidly metabolized in the liver and is excreted in both the urine and feces.

We previously used microarray analyses to show that anesthesia under sevoflurane affected expression ratios of 177 of 10,000 genes in various organs from rats [7]. The highest number of altered genes was detected in the liver and included several genes encoding drug-metabolizing enzymes (DMEs). Furthermore, we investigated the expression ratios of eight genes encoding DMEs that showed the greatest alterations among the affected genes in the liver [5]. We also investigated changes in brain metabolic phenotypes in response to anesthetics by means of a metabolomics study and demonstrated that intravenous and inhalational anesthetics exert differential effects on brain metabolism under anesthesia [8].

Metabolomics is defined as “the exhaustive analysis of endogenous metabolites”. Metabolomics can reveal profiles of endogenous low molecular weight metabolites and can measure dynamic multiparametric responses of living systems to internal and external influences [9]. The main analytical techniques employed for metabolomic studies are based on proton nuclear magnetic resonance (^1^H-NMR) spectroscopic analysis. To interpret ^1^H-NMR digitized spectra data, pattern recognition analysis was used in the present study. One of the most useful and easily applied pattern recognition analyses is principal component analysis (PCA). Moreover, ^1^H-NMR spectroscopy is the only method that can measure many types of metabolites in a tissue or in an organism noninvasively and nondestructively.

The liver plays an important role in drug metabolism, and anesthetics change liver metabolism. Clarification of the different effects of different anesthetics on liver metabolism is important and could provide useful information with regards to the rational selection of anesthetics for clinical use. The purpose of this study was to compare metabolic responses of the liver to anesthesia with representative inhalational (sevoflurane, isoflurane) and intravenous (propofol, dexmedetomidine) anesthetics using pattern recognition ^1^H-NMR spectroscopy with multivariate analyses.

## Methods

**Figure 1:**
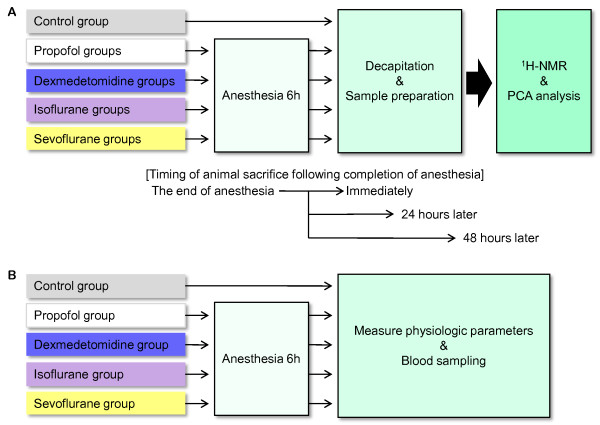
**Flow chart of the experimental protocol. (A)** Experimental protocol of ^1^H-NMR and PC analysis. **(B)** Experimental protocol for measurement of physiologic parameters.

The flow chart of the experimental protocol is shown in Figure 1. A previous study model [8] on acquisition of data, data processing and statistical analysis of ^1^H-NMR data was used.

### Animals and preparation

The animal protocol was approved by the Animal Experimental Ethics Review Committee of Nippon Medical School and involved 9-week-old male Wistar rats weighing 300 ± 20 g (Saitama Experimental Animals Supply, Saitama, Japan). A venous catheter was inserted into the tail vein of all rats, and rats was housed in individual plastic cages (45 × 32 × 23 cm). Rats were anesthetized for 6 h with sevoflurane, isoflurane, propofol or dexmedetomidine, and animals were sacrificed immediately after completion of anesthesia (0-h time-point) or 24 or 48 h later (24 or 48-h time-points). Rats undergoing inhalational anesthesia were placed in plastic boxes supplied with sevoflurane (Maruishi Pharmaceutical, Osaka, Japan; 2.4% gas-air mixture) [10] or isoflurane (Abbot Japan, Tokyo, Japan; 1.5% gas-air mixture) [11] at a rate of 6 L/min (33% oxygen), and normal saline was administered via a venous catheter at a rate of 1 mL/h. Rats undergoing intravenous anesthesia were housed in plastic boxes supplied with 33% oxygen at a rate of 6 L/min and were administered propofol (AstraZeneca, Osaka, Japan; 600 μg/kg/min) [12] or dexmedetomidine (Hospira Japan, Osaka, Japan; 1 μg/kg/min) [13] via a venous catheter at a rate of 1 mL/h. All rats were allowed to breathe spontaneously.

Rats were randomly assigned to 1 of 13 groups (n = 6 per group; one control group and 12 anesthesia groups). Four anesthesia groups (sevoflurane, isoflurane, propofol or dexmedetomidine group) were divided into three subgroups, according to the time of animal sacrifice (immediately after completion of anesthesia or 24 or 48 h later). In the control group, rats were housed in individual plastic cages (45 × 32 × 23 cm) and were not given any anesthetics. These animals were not given access to feed, and they were sacrificed 6 h later. The left lateral lobe of the liver was extracted from each sacrificed rat within 1 min after death, and livers were immediately frozen in liquid nitrogen and stored at −80°C.

### Physiological parameters

Physiological variables were measured in the five separate groups not taken for liver metabolic evaluation (n = 6 each), including those treated with isoflurane, sevoflurane, propofol, dexmedetomidine, or no anesthetic (control group) (Figure 1B). For all rats, a catheter was inserted into the tail vein, and lactated Ringer’s solution was infused continuously at 10 mL/kg/h. During the experiment, each rat was placed into a plastic cage (45 × 32 × 23 cm). All rats were supplied with 33% oxygen and were allowed to breathe spontaneously. Body temperature was maintained at 37°C with a heat lamp. Anesthetics were administered as described above. An arterial catheter was placed in the left femoral artery in all rats to draw blood samples for measurement of arterial PO_2_, arterial PCO_2_, arterial blood pH, plasma glucose concentrations and to monitor heart rate and arterial blood pressure. Anesthesia groups were anesthetized for 6 h. In the control group, rats were placed in a rat tunnel and allowed to recover from anesthesia. Blood samples were taken 2 h later. In the anesthesia groups, blood samples were taken 6 h after the induction of anesthesia.

### Sample preparation

Neutral extraction was performed according to the method described by Yoshioka et al. [14]. The method described by Folch [15] was originally designed for extraction of hydrophobic substances, but this method is also useful for extracting low molecular weight hydrophilic organic compounds, essentially without any in vitro modifications, oxidation hydrolysis or decomposition. Frozen tissue samples were ground and pulverized into a fine powder in liquid nitrogen using a frozen cell crusher (Cryo-Press^TM^, Microtec, Chiba, Japan) and some of the fine powder (approximately 1.0 g) was then homogenized in 10 mL of a chloroform/methanol (2:1) mixture. After removing the residues by filtration, 1 mL of distilled water was then added to the filtrates. After thoroughly mixing, they were left to stand for 24 h at 4°C to separate the hydrophilic phase layer from the hydrophobic phase. The hydrophilic phase was evaporated in an evacuated centrifuge overnight. Dried extracts were reconstituted in 0.6 mL of deuterium oxide (D_2_O) (ISOTEC Inc., Laurenceville, NJ, USA) containing 0.25 mM sodium (3-trimethylsilyl) tetradeuteriopropionate-2,2,3,3-d4 (TMSP) (MSD Isotopes, Montreal, Canada) and then were pipette into 5-mm NMR tubes (Wilmad-LabGlass, Buena, NJ, USA) for subsequent NMR measurements. D_2_O provided a deuterium field frequency lock for the NMR spectrometer, while TMSP provided an internal chemical shift reference (δ = 0.00). The pH of the sample solutions was 7.2 to 7.4.

### Acquisition of proton nuclear magnetic resonance data

Solution state ^1^H NMR spectroscopy was performed at a proton resonance frequency of 300 MHz using an ECX NMR spectrometer interfaced with a TH5 probe (normal geometry, auto tunable type) equipped with an automatic 16-position sample changer and Delta^TM^ NMR processing and control software (version 4.3.2, JEOL Ltd., Tokyo, Japan). Two different ^1^H NMR spectra were acquired for each sample. In all cases, the water resonance was suppressed using a conventional presaturation pulse sequence for the water (HDO) proton signal suppression based on homo-gated irradiation and dante pulse sequence (presaturation time = 2 s, dante pulse = 8 μs, dante interval = 0.1 ms, dante loop = 185, dante attenuator = 24dB). Standard one-dimensional (1D) spectra were acquired using a 45° pulse, 5,580-Hz spectral width, a relaxation delay of 2.0 s between pulses (repetition time = 3.47 s), and 400 transients. Two-dimensional J RES spectra were collected using a double spin echo sequence with 48 transients. This method yields good information on the multiplicity and coupling patterns of resonances, which facilitates molecule identification. The appropriate projection of this spectrum onto the chemical shift axis yields a fingerprint of peaks from only the most highly mobile small molecules, with the added benefit that all of the spin coupling peak multiplicities have been removed.

### Data processing and reduction

The resultant spectra were processed using Alice2^TM^, version 5.5 (JEOL DATUM Ltd., Tokyo, Japan). Free induction decays (FIDs) were subjected to an exponential weighting function of 0.5 Hz, Fourier transformed from the time to frequency domain, and then phased manually, followed by linear baseline correction and referencing to the TMSP singlet at 0.00 ppm.

To simplify the ^1^H NMR spectra of the tissues by means of data compression, all spectra were integrated between 0.5 and 9.5 ppm using an integration macro written within the Alice2 for Metabolome^TM^, version 1.0 (JEOL DATUM Ltd., Tokyo, Japan) software package, which integrated the spectrum into 215 segments (buckets) with 0.04 ppm integral regions. Regions containing resonances of residual water (4.6 ppm to 5.0 ppm) were excluded before integration. Close inspection of the important integral regions for pattern recognition identified some cases where one resonance straddled two or more integral buckets, and therefore, these integral regions were corrected manually combining or extending the regions to include one resonance in a single integral region. Other regions that largely consisted of noise were excluded to produce more significant multivariate mapping. Finally, all the spectral data were reduced into 186 buckets. To account for the bulk mass differences between samples, each spectral region was normalized to the sum of all of the integrals of the buckets.

Assignments of metabolites in the ^1^H NMR spectra were made by comparing the proton chemical shifts with literature values [16,17] and by comparison with spectra of authentic compounds via an in-house spectral database.

### Multivariate analysis and statistical analysis

Calculated results obtained from all measured spectra were exported to a spreadsheet as a text file, which was then used as the input into the pattern recognition/multivariate statistics software package for non-biased metabolic profiling. Datasets were imported into the ADMEWORKS/ModelBuilder^TM^ software, version 4.5 (Fujitsu Kyushu Systems Ltd., Fukuoka, Japan) software package. All data were mean-centered, and a multicollinearity test and genetic-algorithm were used to identify the buckets that included the resonances from key metabolites that differed among the groups. Using the selected bucket data, PCA was performed to discern the presence of inherent similarities between spectral profiles. Data were visualized using the PC score and loading plots. PCA was used to examine trends and clustering in an unsupervised manner. In this analysis, the algorithm calculates the maximum amount of correlated variation in a data set and scores each spectrum according to this variation along PC1. This procedure is repeated for other components until the majority of the variations in the data set are described. Being an unsupervised method, spectra are grouped according to the highest amount of variance in the data set, regardless of the group of each sample. For each type of analysis, data were visualized by plotting PC scores. In the plots, each point represents an individual sample. The plots allow the recognition of clusters of samples with similar scores. Each score plot has an associated loading profile, which helps in identifying the spectral regions (metabolites responsible for the sample clustering observed).

An analysis of variance (ANOVA) test was used to compare physiological variables between the experimental and control groups. P < 0.01 was considered to be statistically significant. Data are given as the mean ± standard deviation (SD).

## Results

### Physiological parameters in control and anesthetized rats

Physiologic parameters were observed in control and anesthetized rats (Table 1). There were no significant differences in pH, PaO2, PaCO2, heart rate, mean arterial pressure or plasma glucose concentration between groups.

**Table 1:** Physiological Parameters in Control and Anesthetized Rats

	**Control**	**Propofol**	**Dexmedetomidine**	**Isoflurane**	**Sevoflurane**
pH	7.45 ± 0.01	7.42 ± 0.02	7.42 ± 0.02	7.41 ± 0.03	7.41 ± 0.02
PaO_2_ (mm Hg)	107.1 ± 7.5	102 ± 10.0	109 ± 8.2	103 ± 10.1	106 ± 6.5
PaCO_2_ (mm Hg)	37.5 ± 1.4	43.5 ± 3.6	39.5 ± 3.7	43.5 ± 2.0	41.5 ± 2.9
HR (beats/min)	301 ± 25	285 ± 25	294 ± 18	292 ± 25	295 ± 24
MAP (mm Hg)	111 ± 6.0	95 ± 10	100 ± 9.0	95 ± 9	97 ± 8.5
GLU (mg/dL)	143 ± 18.0	157 ± 23.0	144 ± 20.7	148 ± 15.6	142 ± 10.8

Data represent mean ± SD for six animals in each group. Low mean blood pressure and high PCO_2_ were observed following the administration of anesthetic drugs. However, there were no significant differences between the anesthetized groups and the control group. HR: heart rate; MAP: mean arterial pressure; and GLU: plasma glucose concentration.

### Proton NMR of hydrophilic liver extracts in control rats

**Figure 2:**
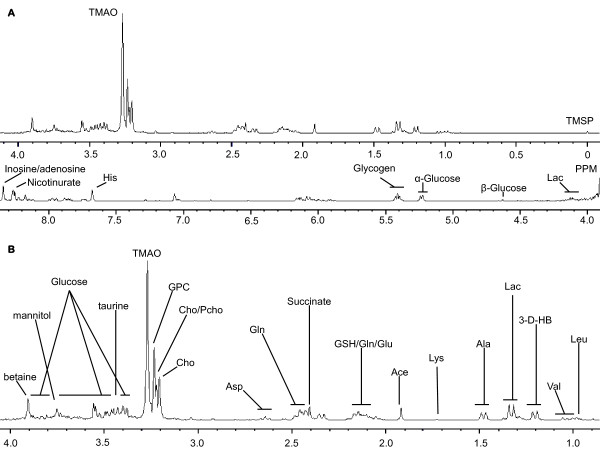
**Proton NMR spectrum of a hydrophilic liver extract in a control rat. (A)** 0.00 ppm to 9.00 ppm and **(B)** 1.00 ppm to 4.00 ppm (Ace: acetate; Ala: alanine; Asp: aspartate; Cho: choline; Gln: glutamine; Glu: glutamate; GPC: glycerophosphorylcholine; GSH: glutathione; His: histidine; Lac: lactate; Lys: lysine; PCho: phosphocholine; TMAO: trimethylamine-N-oxide; Val: valine; and 3-D-HB: 3-D-hydroxybutyrate.).

A typical proton NMR spectrum of hydrophilic extracts from rat livers in the control group is shown in Figure 2. Assignments of metabolites in the ^1^H NMR spectra were made by comparing the proton chemical shifts with literature values [16,17] and by comparison with spectra of authentic compounds via an in-house spectral database.

### PCA score plots

**Figure 3:**
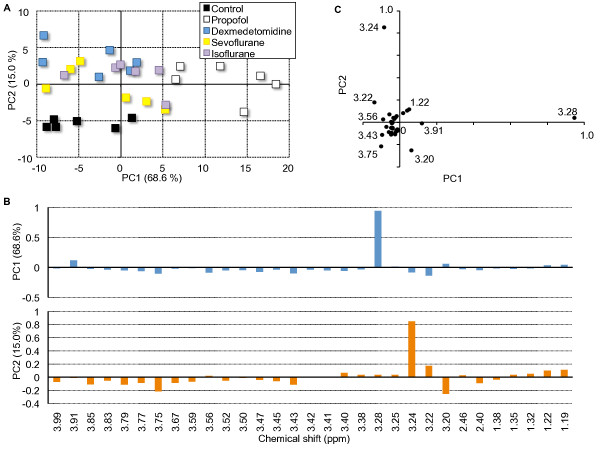
**PCA score plot of hydrophilic liver extracts from the rats sacrificed immediately after anesthesia. (A)** Each plot represents the metabolic data set from an individual rat. Groups are delineated as follows: control (black), propofol (white), dexmedetomidine (blue), sevoflurane (yellow), isoflurane (purple). The plot for each group was clearly divided into separate clusters. In particular, the propofol group was separated from the other groups in the direction of PC1 dominantly. The dexmedetomidine group was separated from the other groups in the direction of PC2. The separation in data clusters was subtler when comparing the inhalational anesthetic groups and the control group. Variables (n = 31 reduced from 186). **(B)** PC loading plot versus chemical shift are graphically illustrated. The numbers indicate the center value of the integral area of each of the conditions as follows: 1.19 and 1.22 ppm (3-D-hydroxybutyrate (3-D-HB)), 1.32 and 1.35 ppm (lactate), 2.40 ppm (succinate), 2.46 ppm (glutamine), 3.20 ppm (choline), 3.22 ppm (choline, phosphocholine), 3.24 ppm (glycerophosphorylcholine (GPC)), 3.28 ppm (trimethylamine-N-oxide (TMAO)), 3.43 ppm (taurine), 3.75 (mannitol), 3.91 (betaine) and 3.30-4.00 ppm (glucose). **(C)** Each plot represents the PC loading plot of individual data sets to distinguish single putative NMR peaks responsible for the clustering pattern observed in the PCA score plot. The 3.28 ppm (TMAO) peak was an effective metabolite used to separate the PC scores in the PC1 direction. The 3.24 ppm (GPC) peak was also effective for separation in the PC2 direction.

A PCA score plot (PC1 vs. PC2) of hydrophilic liver extracts from rats sacrificed immediately after anesthesia is shown in Figure 3A. PCA is a multivariate technique that reduces highly dimensional data into only a few PCs, which are variables created from the linear combinations of the starting variables with appropriate weighting coefficients. The first PC (PC1) contains the largest part of the variance of the data set. In this manner, the data can be reduced into two dimensions, which allows graphic representation of data trends. The plots for the intravenous anesthetic groups (propofol and dexmedetomidine) and inhalational anesthetic groups (isoflurane and sevoflurane) were divided into separate clusters. In particular, the propofol group was separated from the other groups in the direction to PC1 dominantly. The dexmedetomidine group was separated from the other groups in the direction to PC2. By contrast, the separation of data clusters was subtler when comparing the inhalational anesthetic groups and the control group.

### PC loading plots of individual data sets

Loading plots for PC1 vs. PC2 corresponding to Figure 3A are shown in Figure 3B and Figure 3C, respectively. Loading plots show the relative contribution to the PCs of the original variable. The numbers in the loading plots indicate the center value of each of the integral area as follows: 1.19 and 1.22 ppm (3-D-hydroxybutyrate (3-D-HB)), 1.32 and 1.35 ppm (lactate), 2.40 ppm (succinate), 2.46 ppm (glutamine), 3.20 ppm (choline), 3.22 ppm (choline, phosphocholine), 3.24 ppm (glycerophosphorylcholine (GPC)), 3.28 ppm (trimethylamine-N-oxide (TMAO)), 3.43 ppm (taurine), 3.75 (mannitol), 3.91 (betaine) and 3.30-4.00 ppm (glucose). TMAO was most effective metabolite that separated the PC scores in the PC1 direction. GPC was also effective in separation in the PC2 direction.

### PCA score plots over time

**Figure 4:**
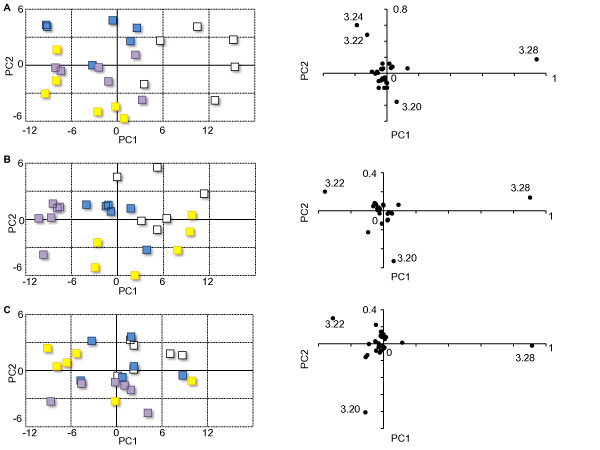
**Time course of PCA score plot.** Graphs located on the left side represent PCA score plots of each group, and graphs on the right side represent PC loading plots corresponding to each graph on the left. Graphs indicate the data from rats sacrificed immediately after anesthesia **(A)**, 24 h after completion of anesthesia **(B)**, and 48 h after completion of anesthesia **(C)**. Variables of each graph are **(A)** n = 31 reduced from 186, **(B)** n = 31 reduced from 186, **(C)** n = 31 reduced from 186. The different marks represent each group: propofol (white), dexmedetomidine (blue), sevoflurane (yellow), and isoflurane (purple). The PCA scores plot revealed that the differences between anesthetized groups decreased with time. The effect of propofol and dexmedetomidine resolved by the 24-h time-point; however, the effect of sevoflurane and isoflurane persisted **(B).**

Changes in liver metabolism were observed over time. The PCA score plot revealed that the differences between anesthetized groups get into trifle with time (Figure 4A-4C). Consequently, liver metabolism was compared among different groups at the 0-h time-point. On the other hand, the PC scores at the 24-h time-point showed that the effect of intravenous anesthetics on the liver resolved quickly when compared to the effect of inhalational anesthetics (Figure 4B).

## Discussion

The present study demonstrated that different liver metabolic phenotypes result from exposure to different types of anesthetic agents. In particular, the liver metabolic phenotype after exposure to propofol was markedly different from that after exposure to other anesthetic agents. However, the differences in liver metabolic phenotype between anesthetics decreased over time.

In this study, experiments were conducted using intravenous anesthetics (propofol and dexmedetomidine) and inhaled anesthetics (isoflurane and sevoflurane). These anesthetics are extensively used in everyday clinical practice. Metabolomic changes in the livers of rats anesthetized with one minimum alveolar concentration of inhaled anesthetics (sevoflurane at 2.4%, isoflurane at 1.5%) [10,11] or with continuous infusion of intravenous anesthetics (propofol at 600 μg/kg/min, dexmedetomidine at 1 μg/kg/min) [12,13] were investigated. Infusion of propofol at 600 μg/kg/min is a median effective dose (ED50) for rats. These doses were chosen because lower doses may not induce adequate anesthesia and because higher doses can produce hemodynamic changes that can independently alter liver metabolism. In a previous study of changes in brain metabolism over time in response to anesthetics, rats were anesthetized for 2 h and at 6 h [8]; the 6-h anesthesia groups exhibited clear metabolic changes. Therefore, in the present study, it was thought that 6 h was a sufficient time period to produce metabolic changes, but sufficiently short to prevent any toxicity.

Separation of PCA scores between the different groups was related to differences in 3-D-HB, choline, phosphocholine, GPC, TMAO, taurine, mannitol, betaine and glucose levels (Figure 3C). 3-D-HB is a ketone body derived from fatty acids metabolism in liver mitochondria (lipid β-oxidation). In the context of hepatic insufficiency, lipid β-oxidation and gluconeogenesis accelerates, and acetyl-coenzyme A (acetyl-CoA) derived from glycolysis and lipid β-oxidation exceeds the capacity of the Krebs cycle [18,19]. Normally, ketone bodies are transported from the liver to other tissues, where they can be reconverted to acetyl-CoA to produce energy; impairment of the Krebs cycle leads to increased release of ketone bodies from the liver as fuel for other tissues [20].

A change in the level of hepatic glucose in anesthetized rats suggests an alteration in the rate of glycogenolysis and glycolysis that is consistent with mitochondrial impairment, and can lead to an inability to use pyruvate in the Krebs cycle and to enhancement of anaerobic metabolism [21]. Thus, the changes of 3-D-HB and glucose likely reflect a general decrease in energy metabolism.

In addition, rats were not fed during this study. Thus, it is possible that changes in the level of glucose, mannitol and taurine may have resulted from the fasting state. Among the four anesthetics administered in this study, only propofol can provide calories due to its fat content. Indeed, propofol is a 1% fat preparation, which corresponds with 1.1 kcal/mL and a total of 7.3 kcal administered to rats during this study. Mannitol is a sugar alcohol, while taurine is related to bile acids and digestion. Thus, the fasting state may also affect levels of those substances.

Choline and phosphocholine are metabolic products of phosphatidylcholine, which is a major membrane constituent. GPC is also a membrane constituent, while TMAO is a product of choline degradation. Betaine helps to maintain cellular osmotic pressure when the cell membrane damaged. Increased levels of choline, phosphocholine, GPC, TMAO and betaine are associated with cell membrane disruption [22].

Changes of endogenous metabolites in hepatic tissue suggest that propofol was the most influential anesthetic on hepatic energy metabolism among the four anesthetics administered in the present study. PC scores showed that TMAO was the most effective metabolite that separated the propofol group from the other groups in the PC1 direction (Figure 3A, 3B,3C). The dexmedetomidine group was separately clustered from the other groups, but the extent of the difference on the PCA plot was smaller than the difference between the propofol group and the other groups. PC scores showed that GPC was effective in separation of the dexmedetomidine group from the other groups in the PC2 direction (Figure 3A,3B,3C). It is possible that the intravenous anesthetics affected the metabolism in the liver by different mechanisms. There was little difference in hepatic metabolites when comparing the inhalational anesthetic groups to the control group. However, the effect of inhalational anesthetics persisted at the 24-h time point while the effect of intravenous anesthetics on the liver resolved over time (Figure 4B), suggesting that propofol and dexmedetomidine were metabolized rapidly when compared with sevoflurane and isoflurane. The loading plot included in Figure 4A-4C remained static over time, and at the 48-h time-point, PCA plots were mixed, and the difference between groups became unclear (Figure 4C).

Sevoflurane and isoflurane are metabolized by hepatic cytochrome P450 CYP2E1 [23], and hepatotoxicity is rarely seen with these two agents [24-27]. In a previous study, there were no significant differences in liver function or total hepatic blood flow when comparing sevoflurane and isoflurane anesthesia [28]. This finding is consistent with observations from the present study, in which PCA scores were relatively similar when comparing the inhalational anesthetic groups and the control group. Indeed, investigators have suggested that liver function is relatively preserved with inhalational anesthetics when compared with intravenous anesthetics [29,30].

Propofol is rapidly metabolized in the liver by conjugation to glucuronide and sulfate to produce water-soluble compounds, and 68.3% of these compounds are excreted in the urine within 24 h [31]. Dexmedetomidine is rapidly distributed and extensively metabolized in the liver. It undergoes conjugation (41%), N-methylation (21%) or hydroxylation followed by conjugation, and 85% of the resulting compounds are excreted in the urine within 24 h [32,33]. Some reports suggest that dexmedetomidine preserves blood flow to the most vital organs (e.g., brain, heart, liver, kidneys) [34] and that there is no difference in clearance of indocyanine green or hepatic blood flow when comparing propofol and dexmedetomidine [32,35]. By contrast, the PCA scores in the present study suggest that propofol has a larger effect on hepatic metabolism when compared with dexmedetomidine.

In previous studies, propofol administration did not affect hepatic arterial blood flow [36], but increase total liver blood flow, portal tributary blood flow, and hepatic oxygen consumption [37]. The increase in oxygen consumption may reflect an increase in hepatic metabolic activity during the clearance of propofol and reflect propofol-induced changes in hepatic metabolism. Previous studies reported that the expression ratios of genes encoding DMEs were elevated under anesthesia and that the expression ratio of Ugt1a7 (a gene encoding DME) was elevated under propofol anesthesia. Ugt1a7 is biotransformation enzyme of the glucuronidation pathway that transforms small lipophilic molecules into water-soluble excretable metabolites [38], and propofol is mainly metabolized by glucuronidation in the liver. In addition, propofol is administered as an emulsion consisting of soybean oil, glycerol, and purified egg phosphatide, which may also affect hepatic metabolism. Further, propofol delivers calories in the form of fat, which may result in differences from other anesthetics and control groups on PCA in this study.

To investigate the utility of ^1^H-NMR for clinical applications, the hydrophilic fraction of the rat liver extracts were analyzed in this study. When the metabolic state is investigated for clinical purposes, the major target is the hydrophilic fraction. The extracted endogenous metabolites in the present study may have utility as a marker of liver metabolism, thereby enhancing the clinical utility of ^1^H-NMR.

Metabolomics is an exhaustive analysis, and we did not attempt to accurately quantitate individual NMR peaks in this study. Our main purpose was to compare the patterns of spectra using chemometric methods to visualize the metabolic phenotypes of the samples. Further investigations that target individual metabolites with refined analysis may be of benefit.

In the present study, metabolite profiling of the liver showed that anesthesia with propofol, dexmedetomidine, sevoflurane and isoflurane exerted different effects on rat liver metabolism. Although analysis of metabolic substances from the liver after exposure to various drugs has been conducted [39], an exhaustive analysis of liver metabolites after exposure to anesthetics has not previously been reported. Thus, the results from this study may enhance the clinical use of anesthetics.

## Conclusions

In conclusion, pattern recognition analysis of ^1^H-NMR of liver tissue under anesthesia revealed that propofol, dexmedetomidine, sevoflurane and isoflurane exert different effects on rat liver metabolism. In particular, liver metabolism was markedly altered after exposure to propofol. The inclusion of fat within the propofol preparation may contribute to this finding. Further, the effects of intravenous anesthetics (propofol, dexmedetomidine) resolved rapidly when compared with the effects of inhalational anesthetics (sevoflurane, isoflurane).

## Abbreviations

DMEs: Drug-metabolizing enzymes; GPC: Glyserophosphorylcholine; H-NMR: Proton nuclear magnetic resonance; PCA: Principal component analysis; 3-D-HB: 3-D-hydroxybutyrate; TMAO: Trimethylamine-N-oxide; TMSP: Tetradeuteriopropionate-2,2,3,3-d4.

## Competing interests

The authors declare that they have no competing interests.

## Authors' contributions

TT and AS conceived and designed the experiments. TT, KH and HK performed the experiments. TT and KH analyzed the data. TT contributed reagents, materials, and analysis tools. TT wrote the paper. All authors read and approved the final manuscript.

## Pre-publication history

The pre-publication history for this paper can be accessed here:

http://www.biomedcentral.com/1471-2342/12/28/prepub
